# Constipation and Cardiovascular Mortality Risk in Patients With Hypertension: A Long-Term Cohort Study

**DOI:** 10.1155/ijhy/9921027

**Published:** 2025-09-25

**Authors:** Junwen Wang, Yuyang Ye, Xuefeng Chen, Xinru Hu, Yong Peng

**Affiliations:** Department of Cardiology, West China Hospital, Sichuan University, 37 Guoxue Street, Chengdu 610041, China

**Keywords:** all-cause mortality, cardiovascular mortality risk, cohort study, constipation, hypertension

## Abstract

**Background:** Whether constipation serves as a risk factor for mortality in hypertensive patients remains an open question. The purpose of the study was to investigate the association of constipation in hypertensive patients with the prognosis for mortality.

**Methods:** The study utilized data from the National Health and Nutrition Examination Survey (NHANES) conducted in 2009-2010 involving hypertensive individuals. Constipation was self-reported over the past 12 months. Cox regression analyses, adjusted for age, sex, and race/ethnicity, were employed to assess the association between constipation and all-cause mortality, as well as cardiovascular mortality. Subgroup and sensitivity analyses were conducted to explore variations in the relationship across different demographic and comorbidity groups.

**Results:** Of the 5199 individuals, 1285 had constipation. Hypertensive patients with constipation exhibited an increased risk of all-cause mortality (HR, 1.40, 95% CI, 0.99 to 1.97, *p*=0.06) and cardiovascular mortality (HR, 1.83, 95% CI, 1.09 to 3.07, *p*=0.02) compared to nonconstipated patients. The Kaplan–Meier survival curves also reflected higher rates of all-cause mortality (92.71% vs. 89.18%, *p* < 0.001) and cardiovascular mortality (97.87% vs. 96.44%, *p*=0.004) in the constipation group. Among hypertensive patients with a PIR ≤ 100, those with constipation exhibited significantly higher all-cause mortality risk than those without (HR 1.95; 95% CI 1.14–2.67; *p* < 0.001). These patients also demonstrated increased cardiovascular mortality risk (HR 1.93; 95% CI 1.12–3.40; *p*=0.019).

**Conclusion:** Constipation shows a significant association with increased cardiovascular mortality risk in hypertensive patients.

## 1. Introduction

The National Health and Nutrition Examination Survey (NHANES) is a nationally representative cross-sectional study that evaluates the health and nutritional status of the U.S. civilian noninstitutionalized population through interviews and physical examinations [1]. Hypertension prevalence among U.S. adults reached 45% [2]. Hypertension is a major public health challenge worldwide, causing systemic damage with high incidence, disability, and mortality rates [3, 4]. Consequently, people suffering from hypertension need to be a primary concern. Cardiovascular diseases significantly contribute to mortality, with hypertension accounting for over half of these deaths [5]. Over the past three decades, the population affected by CVD has almost doubled, and deaths due to CVD have risen by more than 6 million [6]. Even though lifestyle changes and drug treatments have somewhat modified CVD risk factors in those with hypertension, cardiovascular mortality continues to make up one-third of all fatalities [4]. Therefore, it is essential to investigate the risk factors that can be adjusted to lower mortality rates in those with hypertension. Constipation is a condition that affects many people and can be managed or altered [7, 8]. The prevalence of constipation among U.S. adults is approximately 16% [9]. Although often seen as benign, it has a significant impact on the quality of life [10, 11]. Constipation might be linked to hypertension due to the increased blood pressure caused by straining during bowel movements and the mental stress involved [12]. Constipation raises the chances of developing coronary heart disease and experiencing an ischemic stroke [13]. Severe constipation is linked to a heightened risk of atherosclerotic cardiovascular disease [12, 14]. There are only a few studies on the connection between constipation and cardiovascular conditions, and their results have been conflicting [15, 16]. Research shows a significant connection between hypertension, cardiovascular conditions, and constipation [12–14]. However, there is a marked absence of research into the risk of constipation and its connection to cardiovascular mortality and all-cause death among patients with high blood pressure. The uncertain role of constipation as a risk factor for cardiovascular mortality in those with high blood pressure persists. To address this gap, our study focuses on investigating the potential relationship between constipation and the risk of all-cause and cardiovascular mortality among those with hypertension.

## 2. Methods

### 2.1. Study Population

**Figure 1:**
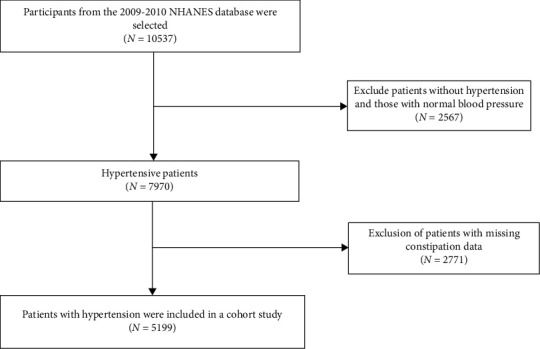
Study flowchart.

The NHANES is a series of cross-sectional complex survey samples of the noninstitutionalized U.S. civilian population, organized by the National Center for Health Statistics [17].A retrospective cohort study design was used in this research. During the NHANES survey cycles from 2009 to 2010, 10,537 participants from the United States were included (Figure 1). After excluding participants without hypertension and those with missing mortality data, 7970 individuals were included in the analysis (Figure 1). After excluding participants without independent variables linked to constipation, the final sample size was 5199 persons (Figure 1).After the Institutional Review Board of the National Center for Health Statistics approved the study protocols, informed consent was collected from all participants.

### 2.2. Definition of Constipation

The question posed to all participants was ‘During the previous 12 months, how often have you had constipation?' The options for responses that participants could choose from included ‘Always,' ‘Most of the time,' ‘Sometimes,' ‘Rarely,' and ‘Never.' The study defined ‘Always,' ‘Most of the time,' and ‘Sometimes' as representing constipation, whereas ‘Rarely' and ‘Never' were defined as no constipation.

### 2.3. Other Variable Definitions

The mobile examination center (MEC) was the site for physical exams and blood sample collection, while participants also engaged in in-home interviews and responded to extra questionnaires. The participants shared self-reported data regarding their age, sex, racial/ethnic background, educational attainment, smoking status, alcohol use, and comorbidities. Race/ethnicity was classified into four groups: White, Black, Mexican, or Others. The classification of smoking status included former, never, and current.

Income was assessed through the income-to-poverty ratio (PIR), determined by dividing the yearly family income by the poverty line, which is adjusted for family size and inflation. To obtain information on a patient's medical history and the drugs they are taking for hypertension, diabetes, hyperlipidemia, coronary heart disease, myocardial infarction, congestive heart failure, stroke, angina, and hyperlipidemia, an interview was conducted at their home. In a physical examination, weight and height were recorded, and the body mass index was determined by dividing the weight in kilograms by the height in meters squared. Collected samples from the MEC were maintained at −20°C until they were analyzed at central laboratories using standard procedures for high-density lipoprotein cholesterol, low-density lipoprotein (LDL) cholesterol, and creatinine. The measurement of LDL cholesterol and glucose was limited to a subsample of participants who had fasted for 8 hours before the survey.

Blood pressure was measured by trained personnel with a mercury sphygmomanometer after the participant sat quietly for at least 5 minutes. Three blood pressure measurements were recorded in this study, and the average of these was used for analysis. The condition of hypertension is identified by having a systolic blood pressure of 140 mmHg or above, a diastolic blood pressure of 90 mmHg or above, or by using blood pressure–lowering medications. The criteria for diabetes included a doctor's diagnosis, an HbA1c level of 6.5% or more, fasting glucose levels of 7.0 mmol/L or higher, random blood glucose levels of 11.1 mmol/L or above, or a two-hour oral glucose tolerance test (OGTT) blood glucose level (mmol/L) of 11.1 or higher, or the use of diabetes medication or insulin.

### 2.4. Follow-Up and Outcomes

All-cause mortality was the primary outcome, with cardiovascular mortality as the secondary outcome up to December 31, 2019. As of December 31, 2019, mortality status was determined through death certificate records linked to the National Death Index, determining the precise cause of death using the ICD-10 classification. Death due to heart diseases, classified under ICD-10 codes I00-I09, I11, I13, and I20–I51, is termed cardiac death. Cardiovascular death encompasses deaths from heart diseases (ICD-10 codes I00-I09, I11, I13, and I20–I51) as well as cerebrovascular diseases (ICD-10 codes I60–I69). The follow-up period for the NHANES MEC was determined from the examination date to either the date of death or the end of the follow-up on December 31, 2019, whichever came first. The median duration of follow-up is 118 months. There is an online link to the final mortality statistics, follow-up times, and underlying leading causes of death (https://ftp.cdc.gov/pub/Health_Statistics/NCHS/datalinkage/linked_mortality/).

### 2.5. Statistical Analysis

Following NHANES analytic guidelines, our analyses incorporated sample weights, clustering, and stratification to ensure the findings are applicable to the entire United States. We applied the correct weights to account for NHANES' complex sampling design, ensuring a representative sample of the U.S. national population. Our study involved weighted multivariate Cox regression analyses where constipation served as the independent variable and mortality was the dependent variable. The purpose of these analyses was to evaluate whether constipation is an independent risk factor for mortality related to CVD or from any cause. The analyses were adjusted to account for age, sex, and race. In addition, we carried out sensitivity analyses across different genders, ethnic groups, educational backgrounds, income levels, and comorbidities.

The statistical analyses were executed using R Version 4.1.3 (R Foundation for Statistical Computing, Vienna, Austria), with a two-tailed *p* value of less than 0.05 regarded as statistically significant. A Kaplan–Meier survival curve was drawn using the ‘ggsurvplot' tool from the R ‘ggplot' library along with the ‘survfit' function from the survival library.

## 3. Results

### 3.1. Patient Characteristics

Our study included 5199 individuals (Table 1). Among them, 1285 individuals had constipation and 3914 individuals did not. The average age of the no constipation group was 46.67 years, which was lower than the constipation group with an average age of 49.35 years (*p* < 0.0001). The no constipation group had a higher percentage of male individuals at 54.13% than the constipation group with 33.41%. The average blood glucose in the no constipation group (5.80 mmol/L) was not significantly different from the constipation group (5.74 mmol/L, *p*=0.36). Similarly, there were no significant differences between the no constipation and constipation groups in high-density lipoprotein cholesterol (1.37 vs. 1.38 mmol/L, *p*=0.72) and LDL cholesterol (3.03 vs. 2.96 mmol/L, *p*=0.17). Additionally, there were no differences in body mass index, systolic blood pressure, diastolic blood pressure, coronary heart disease, myocardial infarction, hyperlipidemia, stroke, angina, and smoking between the constipation and nonconstipation groups. The proportion of laxative use was 5.13% in the nonconstipation group, 26.35% in the constipation group, and 9.87% in the overall population.

### 3.2. Constipation and Mortality Risk in Hypertensive Patients

**Figure 2:**
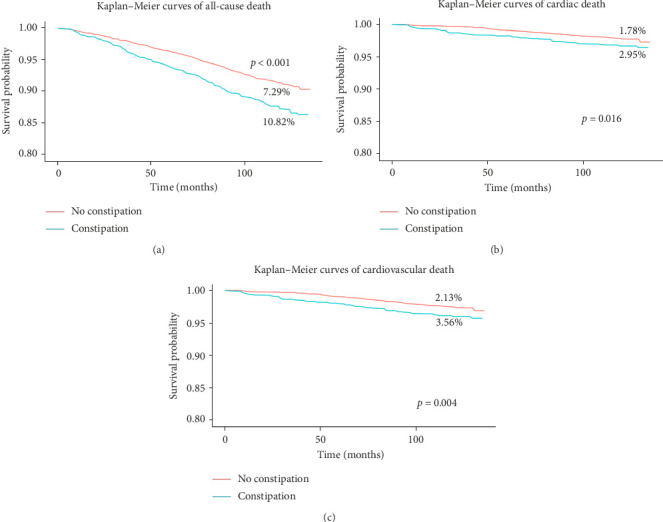
Kaplan–Meier survival curves for incident of all-cause death (a), cardiac death (b), and cardiovascular death (c).

Kaplan–Meier survival curves indicate that all-cause mortality were higher in the constipation group (10.82% vs. 7.29%, *p* < 0.001), cardiac mortality (2.95% vs. 1.78%, *p* = 0.016), and cardiovascular mortality (3.56% vs. 2.13%, *p* = 0.004) than in the nonconstipation group (Figure 2). In the unadjusted models, for all-cause mortality, the hazard ratio (HR) was 1.49 (95% CI 1.19–1.86; *p* < 0.001). For cardiac-specific mortality, the HR was similarly elevated at 1.53 (95% CI 1.14–2.06; *p* = 0.001). Similarly, for cardiovascular mortality, the HR was 1.57 (95% CI 1.19–2.06; *p* = 0.005). After adjusting for age, sex, and race/ethnicity, hypertensive patients with constipation had an increased risk of all-cause mortality (HR, 1.36, 95% CI, 1.08 to 1.71, *p* = 0.01) compared to nonconstipated patients (Table 2). Similarly, after adjusting for age, sex, and race/ethnicity, hypertensive patients with constipation showed an elevated risk of cardiac mortality (HR, 1.40, 95% CI, 1.02 to 1.91, *p* = 0.03) compared to nonconstipated patients (Table 2). The risk of cardiovascular mortality in hypertensive patients with constipation remained increased (HR, 1.39, 95% CI, 1.04 to 1.85, *p* = 0.03) after adjusting for age, sex, and race/ethnicity (Table 2). After controlling for variables including sex, age, ethnicity/race, systolic and diastolic blood pressure, LDL cholesterol, HDL cholesterol, triglycerides, body mass index (BMI), smoking, and alcohol consumption, the adjusted HR for all-cause mortality was 1.40 (95% confidence interval [CI]: 0.99–1.97; *p* = 0.06) (Table 2). The adjusted HR for cardiac mortality was 2.01 (95% CI: 1.07–3.77; *p* = 0.03), and the adjusted HR for cardiovascular mortality was 1.83 (95% CI: 1.09–3.07; *p* = 0.02) (Table 2).

### 3.3. Subgroup and Sensitivity Analyses

**Figure 3:**
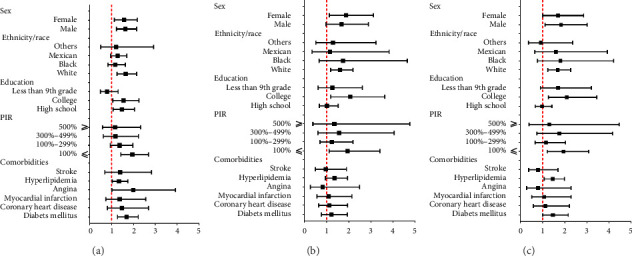
Forest plot of stratified analyses of the all-cause mortality (a), cardiac death (b), and cardiovascular death (c). PIR: poverty income ratio.

Subgroup analyses were conducted to examine whether the association between constipation and all-cause mortality, cardiac mortality, and cardiovascular mortality varied based on baseline demographic characteristics and comorbidities. Stratification was performed according to baseline sex, race/ethnicity, educational level, PIR level, diabetes, hyperlipidemia, coronary heart disease, myocardial infarction, congestive heart failure, stroke, angina, and hyperlipidemia (Figure 3 and STable 1–3). In hypertensive patients with a PIR of ≤ 100, the risk of all-cause mortality is higher in those with constipation compared to those without constipation (HR 1.95 95% CI 1.14 to 2.67, *p* < 0.001) (Figure 3(a) and STable 1). Additionally, they have a higher risk of cardiovascular mortality (HR 1.93, 95% CI 1.12 to 3.40, *p*=0.019) (Figure 3(c) and STable 3).

## 4. Discussion

Our study revealed a higher risk of cardiac mortality and cardiovascular mortality in hypertensive patients with constipation compared to nonconstipated patients. Sensitivity analysis indicated an elevated risk of mortality in constipated hypertensive patients with a PIR ≤ 100%.

Previous studies have utilized NHANES data to examine the relationship between constipation and conditions such as depression, as well as potential etiological factors of constipation [18–20]. However, research on the impact of constipation on mortality risk remains limited, and the association between constipation and mortality among hypertensive patients warrants further investigation. We found that constipation is an independent risk factor for mortality among hypertension. Adults of diverse ages, genders, and definitions of constipation experience constipation at a rate between 3% and 79%, depending on their age, sex, and diagnosis [21, 22]. The fundamental goal of treating hypertensive patients is to reduce the risk of mortality [23, 24]. Previous studies have found an association between constipation and the risk of mortality of ischemic stroke [25]. While previous studies have explored the relationship between constipation and cardiovascular mortality, the results have been contradictory and have not specifically investigated hypertensive patients [15, 16]. Constipation can lead to elevated blood pressure, which may be one of the reasons for the increased cardiovascular mortality in constipated patients [12]. However, our study revealed no significant difference in systolic and diastolic blood pressure between both groups. This implies that constipation independently increases the risk of all-cause and cardiovascular mortality, regardless of blood pressure levels.

The association between the risk of mortality and constipation may be intricately linked through the gut microbiota [26, 27]. The association between gut microbiota and atherosclerotic cardiovascular diseases has been confirmed in previous studies [28]. Gut microbiota imbalance is associated with various cardiovascular diseases [29]. Constipated patients may experience a shift in gut microbiota leading to inflammation and systemic inflammatory responses [30]. Chronic constipation patients are often in a state of chronic inflammation, which is considered a significant factor promoting atherosclerosis [30, 31]. The gut microbiota is associated with constipation, a condition that may reflect altered gut microbiota. Thus, alterations in gut microbiota could contribute to the increased mortality risk in patients, although further research is needed. Disruptions in autonomic balance might contribute to gastrointestinal issues such as constipation [32]. Chronic constipation may lead to prolonged activation of sympathetic responses, further exacerbating autonomic imbalance [32]. Given the potential bidirectional relationship between constipation and autonomic dysfunction, lifestyle interventions targeting both gastrointestinal health and autonomic balance may hold promise in reducing cardiovascular risk [33, 34]. Dysregulation of the autonomic nervous system might be implicated in constipation pathogenesis; however, further studies are necessary to establish causality. Further research is warranted to explore the effectiveness of interventions such as dietary modifications, physical activity, and stress reduction in ameliorating constipation in hypertensive patients [34–36]. This could also be one of the ways to reduce the risk of mortality in hypertensive patients. While our findings demonstrate an association between constipation and elevated mortality risk, the potential for reverse causation must be considered. Several conditions associated with premature mortality (e.g., diabetes mellitus and Parkinson's disease) demonstrate strong associations with constipation [37, 38]. Therefore, the observed mortality risk might reflect underlying constipation-inducing comorbidities instead of a direct effect of constipation itself. Furthermore, Parkinson's disease, diabetes, and malignancies are conditions that may independently contribute to both constipation and increased mortality, thereby potentially confounding the observed association between constipation and mortality. Further research is warranted to clarify these relationships.

Interestingly, there might be a higher risk of mortality in hypertensive patients with PIR ≤ 100%. The relationship between economic and educational disparities and cardiovascular diseases may stem from variations in behavior, psychological factors, lifestyle, and living environments [39–41]. Our study found that the higher mortality risk in patients with low-income levels may be attributed to delayed treatment and a lack of disease rehabilitation [42]. The interconnection between income and educational levels may contribute to cardiovascular events through various pathways [43]. Low-income levels remain a significant risk factor for cardiovascular diseases regardless of educational attainment [44]. In our study, sensitivity analysis revealed differences in various groups. The lack of statistical significance in some subgroup analyses may be attributed to the relatively small sample sizes, warranting further investigation for confirmation.

This study demonstrates that comorbid constipation is associated with increased mortality risk in hypertensive patients, a finding with important clinical implications. Constipation could serve as a practical biomarker for cardiovascular risk stratification in hypertension management. Clinicians should therefore (1) monitor bowel habits regularly, (2) adjust drug regimens to minimize constipation (particularly avoiding opioid receptor antagonists and Histamine 1 receptor antagonist when appropriate), and (3) recommend dietary fiber increase and regular physical activity to improve both gastrointestinal and cardiovascular health [45–47]. Clinical guidelines recognize constipation as a modifiable risk factor in patients with hypertension, indicating that effective management of constipation may help lower cardiovascular mortality risk in this population. Consequently, hypertension management guidelines should include recommendations for the proactive identification and treatment of constipation in hypertensive patients.

From a public health perspective, better constipation management may prevent a significant number of hypertension-related cardiovascular deaths, offering a cost-effective intervention approach. We propose (1) including constipation screening in hypertension guidelines, (2) incorporating bowel health assessments into community healthcare, and (3) developing public education programs about the gut-cardiovascular connection. These findings provide new perspectives for cardiovascular prevention by highlighting the need to (1) study how treating constipation affects cardiovascular outcomes and (2) develop specific strategies for high-risk groups such as elderly hypertensive patients.

### 4.1. Limitations

Firstly, our constipation information was gathered through questionnaire surveys, inquiring about constipation status in the past 12 months, providing a certain level of data stability. However, this approach may introduce recall bias and information bias. Recall bias could result in misclassification of constipation exposure. Potential underreporting of constipation history among deceased patients or their relatives could attenuate the observed constipation-mortality association, whereas overreporting stemming from heightened attention might artificially inflate this relationship. Nondifferential misclassification typically increases random error; however, when the bias direction is balanced between groups, it primarily reduces statistical power without substantially changing the effect direction. The present study employed weighted data representative of the entire US population, thus minimizing recall bias impacts; nevertheless, its potential influence should be acknowledged. Secondly, our findings were based on the American population and require additional validation through further assessments. Nevertheless, all our patients were sourced from the NHANES database, which employs a scientifically sound sampling method. We used sampling weights in our statistical analysis, rendering these data effectively representative of the US population. Furthermore, our study endpoints encompassed all-cause mortality, cardiovascular mortality, and cardiovascular and cerebrovascular mortality. Therefore, the conclusions cannot be extrapolated to specific causes of death, such as those related to heart failure or ischemic heart disease. Lastly, due to data limitations, information on the use of specific medications that may contribute to constipation was unavailable. As a result, some potential confounding factors, including medication use, may not have been fully accounted for in our analysis.

## 5. Conclusion

Constipation is significantly associated with an increased risk of cardiovascular mortality in patients with hypertension. This study provides evidence that constipation is an independent risk factor for mortality in hypertensive patients. The findings emphasize the importance of recognizing and addressing constipation in hypertensive populations for enhanced outcomes.

## Figures and Tables

**Table 1:** Baseline characteristics stratified by constipation status.

Variable	Total (*n* = 5199)	No constipation (*n* = 3914)	Constipation (*n* = 1285)	*p* value
Sex, *n* (%)				< 0.001
Female	2616 (50.50)	1795 (45.87)	821 (66.59)	
Male	2583 (49.50)	2119 (54.13)	464 (33.41)	
Age, years	47.27 (0.52)	46.67 (0.57)	49.35 (0.61)	< 0.001
Glucose, (mmol/L)	5.78 (0.05)	5.80 (0.06)	5.74 (0.06)	0.36
Creatinine (μmol/L)	78.92 (0.67)	79.39 (0.73)	77.26 (1.16)	0.1
HDL cholesterol,(mmol/L)	1.37 (0.01)	1.37 (0.01)	1.38 (0.02)	0.72
LDL cholesterol,(mmol/L)	3.02 (0.03)	3.03 (0.04)	2.96 (0.03)	0.17
BMI, (kg m^2^)	28.85 (0.13)	28.79 (0.16)	29.08 (0.22)	0.31
SBP, (mmHg)	120.20 (0.50)	120.28 (0.52)	119.91 (0.60)	0.46
DBP, (mmHg)	69.37 (0.66)	69.50 (0.69)	68.92 (0.62)	0.16
Comorbidities, *n* (%)				
Coronary heart disease	215 (3.23)	144 (3.12)	71 (3.63)	0.32
Myocardial infarction	231 (3.50)	161 (3.32)	70 (4.16)	0.15
Congestive heart failure	140 (1.98)	92 (1.81)	48 (2.57)	0.08
Stroke	206 (2.94)	148 (2.82)	58 (3.37)	0.43
Angina	134 (1.99)	84 (1.81)	50 (2.63)	0.21
Hyperlipidemia	3776 (71.50)	2810 (70.43)	966 (75.20)	0.05
Smoking, *n* (%)				0.51
Former	1297 (24.90)	958 (24.72)	339 (25.51)	
Never	2776 (55.03)	2113 (55.67)	663 (52.79)	
Now	1126 (20.07)	843 (19.61)	283 (21.70)	
Ethnicities/race, *n* (%)				0.01
Black	907 (10.85)	678 (10.47)	229 (12.16)	
Mexican	950 (8.54)	671 (8.06)	279 (10.20)	
Others	774 (11.02)	570 (10.73)	204 (12.03)	
White	2568 (69.59)	1995 (70.74)	573 (65.61)	
Education, *n* (%)				0.002
College	2553 (58.58)	2005 (60.37)	548 (52.85)	
High school	2036 (35.42)	1486 (34.22)	550 (39.90)	
Less than 9th grade	600 (5.81)	415 (5.41)	185 (7.25)	
Drinking, *n* (%)				0.001
No	2797 (50.59)	2011 (48.52)	786 (57.78)	
Yes	2402 (49.41)	1903 (51.48)	499 (42.22)	
Laxative, *n* (%)				0.001
No	4613 (90.13)	3679 (94.87)	934 (73.65)	
Yes	584 (9.87)	233 (5.13)	351 (26.35)	

Abbreviations: BMI, body mass index; DBP, diastolic blood pressure; HDL, high-density lipoprotein; LDL, low-density lipoprotein; SBP, systolic blood pressure.

**Table 2:** The adjusted hazard ratios (95% CI) of cumulative events by constipation.

Outcome	Model 1^∗^		Model 2^†^		Model 3^‡^	
	HR (95% CI)	*p* value	HR (95% CI)	*p* value	HR (95% CI)	*p* value
All-cause death	1.49 (1.19, 1.86)	< 0.001	1.36 (1.08, 1.71)	0.01	1.40 (0.99, 1.97)	0.06
Cardiac death	1.53 (1.14, 2.06)	0.001	1.40 (1.02, 1.91)	0.03	2.01 (1.07, 3.77)	0.03
Cardiovascular death	1.57 (1.19, 2.06)	0.005	1.39 (1.04, 1.85)	0.03	1.83 (1.09,3.07)	0.02

*Note:* HR calculated with Cox proportional hazard model.

Abbreviations: CI, confidence interval; HR, hazard ratio.

^∗^Unadjusted.

^†^Adjusted for sex, age, and ethnicity/race.

^‡^Model 2 with additional adjustment for systolic blood pressure, diastolic blood pressure, LDL cholesterol (mmol/L), HDL cholesterol (mmol/L), triglyceride (mmol/L), body mass index (kg/m^2^), smoking, and drinking.

## Data Availability

The data that support the findings of this study are available from the corresponding author upon reasonable request.

## Supporting Information

STable 1: Stratified analyses of the all-cause death.

STable 2: Stratified analyses of the cardiac disease death.

STable 3: Stratified analyses of the cardiovascular death.
