# Supplementary material for: Constipation and Cardiovascular Mortality Risk in Patients With Hypertension: A Long-Term Cohort Study
Source: Int J Hypertens. 2025 Sep 25;2025:9921027. doi: 10.1155/ijhy/9921027 (PMC12490922; doi:10.1155/ijhy/9921027)
Supplement: Supporting Information — Additional supporting information can be found online in the Supporting Information section. [file 9921027.f1.docx]

| Character | HR (95% CI) | P | P for interaction |
| --- | --- | --- | --- |
| Sex |  |  | 0.839 |
| Male | 1.614(1.221,2.135) | <0.001 |  |
| Female | 1.560(1.122,2.169) | 0.008 |  |
| Ethnicities/Race |  |  | 0.372 |
| White | 1.631(1.248,2.131) | <0.001 |  |
| Black | 1.159(0.830,1.619) | 0.386 |  |
| Mexican | 1.269(0.951,1.692) | 0.105 |  |
| Other | 1.202(0.496,2.913) | 0.684 |  |
| Education |  |  | 0.19 |
| High School | 1.471(1.062,2.038) | 0.020 |  |
| College | 1.537(1.054,2.242) | 0.026 |  |
| Less Than 9th Grade | 0.792(0.484,1.295) | 0.352 |  |
| PIR |  |  | 0.494 |
| 100%≤ | 1.947(1.420,2.671) | <0.0001 |  |
| 100%-299% | 1.353(0.933,1.962) | 0.111 |  |
| 300%-499% | 1.169(0.614,2.225) | 0.635 |  |
| 500%≥ | 1.155(0.576,2.316) | 0.686 |  |
| Diabetes Mellitus |  |  | 0.045 |
| no | 1.273(0.982,1.650) | 0.069 |  |
| yes | 1.673(1.266,2.211) | <0.001 |  |
| Coronary Heart Disease |  |  | 0.994 |
| no | 1.456(1.169,1.814) | <0.001 |  |
| yes | 1.468(0.806,2.674) | 0.210 |  |
| Myocardial Infarction |  |  | 0.782 |
| no | 1.469(1.168,1.846) | 0.001 |  |
| yes | 1.364(0.729,2.551) | 0.332 |  |
| Angina |  |  | 0.436 |
| no | 1.409(1.088,1.826) | 0.009 |  |
| yes | 1.983(1.005,3.912) | 0.048 |  |
| Hyperlipidemia |  |  | 0.14 |
| no | 2.092(1.283,3.409) | 0.003 |  |
| yes | 1.329(1.021,1.730) | 0.034 |  |
| Stroke |  |  | 0.849 |
| no | 1.481(1.196,1.833) | <0.001 |  |
| yes | 1.389(0.685,2.818) | 0.363 |  |

STable 1 Stratified analyses of the all-cause death

STable2 Stratified analyses of the cardiac disease death

| Character | HR (95% CI) | p | P for interaction |
| --- | --- | --- | --- |
| Sex |  |  | 0.788 |
| Male | 1.671(0.968,2.883) | 0.065 |  |
| Female | 1.874(1.126,3.119) | 0.016 |  |
| Ethnicities/Race |  |  | 0.947 |
| White | 1.608(1.178,2.196) | 0.003 |  |
| Black | 1.746(0.657,4.636) | 0.263 |  |
| Mexican | 1.153(0.348,3.822) | 0.816 |  |
| Other | 1.284(0.510,3.229) | 0.596 |  |
| Education |  |  | 0.105 |
| High School | 1.008(0.671,1.515) | 0.969 |  |
| College | 2.071(1.184,3.622) | 0.011 |  |
| Less Than 9th Grade | 1.263(0.612,2.608) | 0.528 |  |
| Poverty Income Ratio |  |  | 0.857 |
| 100%≤ | 1.947(1.115,3.400) | 0.019 |  |
| 100%-299% | 1.237(0.697,2.194) | 0.467 |  |
| 300%-499% | 1.562(0.603,4.047) | 0.358 |  |
| 500%≥ | 1.351(0.385,4.744) | 0.639 |  |
| Diabetes Mellitus |  |  | 0.465 |
| no | 1.605(0.994,2.592) | 0.053 |  |
| yes | 1.214(0.762,1.933) | 0.414 |  |
| Coronary Heart Disease |  |  | 0.131 |
| no | 1.643(1.205,2.240) | 0.002 |  |
| yes | 1.119(0.644,1.942) | 0.691 |  |
| Myocardial Infarction |  |  | 0.378 |
| no | 1.549(1.100,2.179) | 0.012 |  |
| yes | 1.101(0.564,2.147) | 0.779 |  |
| Angina |  |  | 0.211 |
| no | 1.609(1.184,2.188) | 0.002 |  |
| yes | 0.821(0.271,2.490) | 0.728 |  |
| Hyperlipidemia |  |  | 0.239 |
| no | 2.323(1.122,4.808) | 0.023 |  |
| yes | 1.354(0.943,1.943) | 0.100 |  |
| stroke |  |  | 0.195 |
| no | 1.619(1.164,2.250) | 0.004 |  |
| yes | 0.962(0.487,1.899) | 0.911 | 0.947 |

STable3 Stratified analyses of the cardiovascular death

| Character | HR(95% CI) | P | P for interaction |
| --- | --- | --- | --- |
| Sex |  |  | 0.841 |
| Male | 1.829(1.114,3.004) | 0.017 |  |
| Female | 1.688(0.999,2.851) | 0.050 |  |
| Ethnicities/Race |  |  | 0.612 |
| White | 1.675(1.240,2.262) | <0.001 |  |
| Black | 1.796(0.767,4.207) | 0.178 |  |
| Mexican | 1.594(0.648,3.917) | 0.310 |  |
| Other | 0.916(0.357,2.351) | 0.855 |  |
| Education |  |  | 0.056 |
| High School | 0.972(0.668,1.415) | 0.883 |  |
| College | 2.090(1.269,3.443) | 0.004 |  |
| Less Than 9th Grade | 1.697(0.899,3.202) | 0.103 |  |
| Poverty Income Ratio |  |  | 0.725 |
| 100%≤ | 1.933(1.216,3.072) | 0.005 |  |
| 100%-299% | 1.154(0.657,2.026) | 0.618 |  |
| 300%-499% | 1.753(0.741,4.152) | 0.202 |  |
| 500%≥ | 1.304(0.383,4.445) | 0.671 |  |
| Diabetes Mellitus |  |  | 0.969 |
| no | 1.475(0.951,2.287) | 0.083 |  |
| yes | 1.465(0.998,2.150) | 0.051 |  |
| Coronary Heart Disease |  |  | 0.19 |
| no | 1.675(1.288,2.178) | <0.001 |  |
| yes | 1.127(0.576,2.208) | 0.726 |  |
| Myocardial Infarction |  |  | 0.304 |
| no | 1.607(1.194,2.163) | 0.002 |  |
| yes | 1.074(0.505,2.282) | 0.853 |  |
| Angina |  |  | 0.158 |
| no | 1.654(1.222,2.238) | 0.001 |  |
| yes | 0.791(0.274,2.281) | 0.664 |  |
| Hyperlipidemia |  |  | 0.479 |
| no | 1.964(0.981,3.930) | 0.057 |  |
| yes | 1.455(1.071,1.977) | 0.016 |  |
| Stroke |  |  | 0.059 |
| no | 1.719(1.284,2.301) | <0.001 |  |
| yes | 0.791(0.371,1.689) | 0.545 | 0.612 |
